# Targeting the Immune Network in Endometriosis: A Comprehensive Review of Pathogenesis, Immunomodulation, and Emerging Therapies

**DOI:** 10.3390/ph19071074

**Published:** 2026-07-12

**Authors:** Yukun Mo, Meiye Li, Wei Ye, Zhuozhen Jiang, Xianhua Lin, Kewang Luo

**Affiliations:** 1 Shenzhen Key Laboratory of Birth Defect Prevention and Control, Shenzhen Maternity and Child Healthcare Hospital, Women and Children’s Medical Center, Southern Medical University, Shenzhen 518000, China; falsehood159357@gmail.com (Y.M.); meiyeli2023@gmail.com (M.L.); yeyewei2023@gmail.com (W.Y.); 2 School of Pharmaceutical Sciences, Southern Medical University, Guangzhou 510515, China; 3 School of Stomatology, Southern Medical University, Guangzhou 510515, China; zhuozhen025@gmail.com

**Keywords:** endometriosis, microenvironment, immune cells, immunotherapies, neutrophils, mast cells

## Abstract

Endometriosis is a chronic inflammatory disease characterized by the ectopic growth of endometrial-like tissue and is closely associated with pain and infertility. This review summarizes current evidence on immune dysregulation and emerging immunomodulatory therapies in endometriosis. Relevant clinical and mechanistic studies were identified through a structured literature review and qualitatively synthesized. Increasing evidence suggests that immune dysregulation plays a critical role in disease pathogenesis. Within the immune microenvironment, innate and adaptive immune cells—including macrophages, dendritic cells, neutrophils, mast cells, T cells, and B cells—collectively promote inflammation, angiogenesis, fibrosis, and immune evasion. Emerging immunotherapeutic strategies, including macrophage repolarization, NK cell restoration, dendritic cell modulation, neutrophil extracellular traps inhibition, immune checkpoint blockade, and cytokine/chemokine targeting, have shown promising preclinical and early clinical potential. However, disease heterogeneity, reproductive safety concerns, and the lack of validated biomarkers remain major barriers to clinical translation, highlighting the potential of immunomodulatory therapies as future non-hormonal treatment strategies.

## 1. Introduction

**Figure 1:**
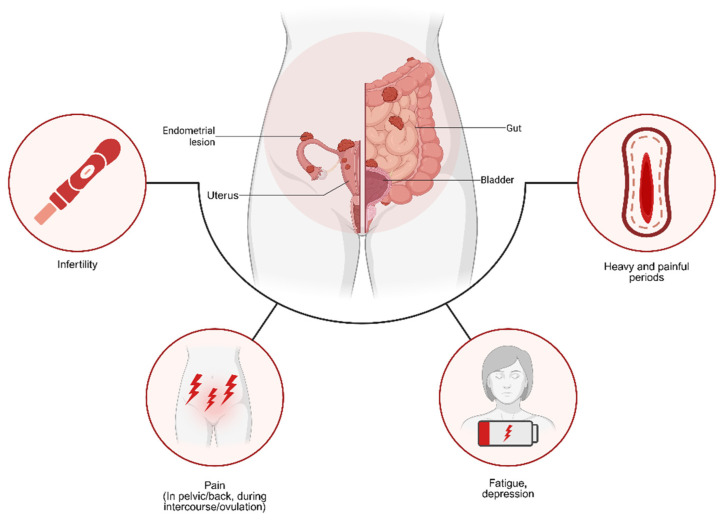
Clinical manifestations and anatomical distribution of endometriosis. Schematic illustration of endometriotic lesion distribution within the pelvic peritoneum, including involvement of the uterus, ovaries, bowel, and bladder. Ectopic endometrial lesions are depicted at multiple anatomical sites. The associated clinical manifestations are summarized, including infertility, dysmenorrhea with heavy menstrual bleeding, chronic pelvic or back pain (particularly during intercourse or ovulation), and systemic symptoms such as fatigue and depression. The pelvic peritoneum is the lower region of the peritoneal cavity. The peritoneal cavity is contained in the abdominopelvic cavity, is bound by the peritoneum, and wraps around organs contained in the abdominopelvic cavity (see [https://www.cancer.gov/publications/dictionaries/cancer-terms/def/peritoneal-cavity] accessed on 6 July 2026).

Endometriosis is a common gynaecological condition among women of childbearing age, affecting 5–10% of women of childbearing age worldwide and imposing a significant economic burden [1]. Endometriosis is a chronic, estrogen-dependent inflammatory disorder characterized by the ectopic implantation of endometrial-like tissue, affecting approximately 10% of women of reproductive age worldwide [2]. The prevalence is markedly higher among specific subpopulations, reaching up to 70% in those suffering from chronic pelvic pain [3]. Recent global estimates based on the Global Burden of Disease (GBD) 2021 study indicate that approximately 22 million women are affected globally, with an age-standardized incidence rate of 162.7 cases per 100,000 population [4,5]. The disease predominantly occurs in women aged 20–29 years, highlighting its significant impact during peak reproductive years. Although age-standardized incidence and prevalence rates have shown a declining trend over the past decades, the absolute number of cases continues to rise due to population growth and improved diagnostic awareness. Notably, substantial geographic and socioeconomic disparities persist, with a higher disease burden observed in low-sociodemographic index regions, likely reflecting differences in healthcare accessibility, diagnostic capacity, and reproductive patterns [4,6]. Importantly, the true prevalence of endometriosis is likely underestimated, as definitive diagnosis often requires laparoscopic confirmation and a considerable proportion of patients remain asymptomatic or experience significant diagnostic delays, typically ranging from 6 to 10 years [7]. Endometriosis is characterized by the growth of endometrial-like tissue outside the uterine lining (endometrium), such as on the peritoneum, ovaries, cervix, exterior of the uterus, in the gut, and on the bladder, as illustrated in Figure 1 [8]. More than half of patients with endometriosis experience abdominopelvic pain as well as more widespread pain manifestations, including chronic back pain, fibromyalgia, dyspareunia, vulvodynia, and migraine [9]. At the same time, the condition presents with gastrointestinal symptoms similar to those of irritable bowel syndrome, which is one of the reasons why it is often misdiagnosed [10,11].

For a considerable period, the pathogenesis of endometriosis has been predominantly explained by Sampson’s theory of retrograde menstruation. This theory proposes that menstrual debris containing viable endometrial cells refluxes through the fallopian tubes into the peritoneal cavity, where these cells subsequently adhere to and implant on pelvic structures [12]. However, retrograde menstruation is a common physiological phenomenon, and only approximately 10% of women develop endometriosis; therefore, this theory alone is insufficient to fully explain the pathogenesis of the disease. In addition, endometriosis can significantly impair female fertility by damaging ovarian tissue, disrupting follicular development, compromising oocyte quality and fertilization, reducing embryo quality and developmental potential, and increasing the risk of pregnancy-related complications, thereby markedly affecting patients’ occupational functioning and quality of daily life [13].

With ongoing research, endometriosis is now recognized as a multifactorial disease, in which alterations in the immune microenvironment, along with genetic, endocrine, inflammatory, and hormonal factors, collectively contribute to the initiation and maintenance of ectopic lesions. Recent genome-wide association studies GWAS have identified some susceptibility loci associated with endometriosis, many of which regulate genes involved in immune responses, inflammatory signaling, tissue remodeling, and hormonal regulation [10]. A more recent multi-ancestry GWAS integrating transcriptomic, epigenetic, and proteomic analyses further expanded the number of susceptibility loci and demonstrated that many candidate genes participate in immune regulation, inflammation, and tissue remodeling [14]. Immune-related susceptibility genes, such as IL1A, have been associated with endometriosis risk, supporting a genetic contribution to inflammatory dysregulation [15]. Earlier GWAS identified susceptibility loci near WNT4, GREB1, and VEZT, providing the first robust evidence that inherited genetic variants contribute to endometriosis development [16]. These pathogenic mechanisms do not act in isolation but instead constitute an integrated and interconnected network with extensive crosstalk [17]. Retrograde menstruation refers to the reflux of menstrual debris containing endometrial cells through the fallopian tubes into the peritoneal cavity, where these cells may adhere to and implant on pelvic structures, whereas factors such as genetic susceptibility and hormonal regulation determine their survival. Critically, immune dysfunction acts as a permissive factor that enables ectopic implants to evade immune clearance, establish vascularization, and progress to clinically significant disease. In recent years, accumulating evidence has demonstrated that various immune cell populations undergo adaptive phenotypic and functional changes in response to the chronic inflammatory stimuli associated with ectopic lesions. Dysregulation of the immune microenvironment is therefore considered a key factor in the establishment and maintenance of endometriotic lesions [18]. The immune microenvironment in endometriosis is complex and dynamic throughout disease progression. Cells from both the innate and adaptive immune systems are present within endometriotic lesions and actively contribute to immunopathological alterations. Immune abnormalities associated with endometriosis include the accumulation of disease-associated macrophages (DAMs), reduced NK cell activity, impaired dendritic cell maturation, and suppressed T cell cytotoxicity [19].

In this review, we discuss the roles of various immune cell populations in endometriosis, as well as potential immunotherapeutic targets within the lesion-associated immune microenvironment. To provide a comprehensive overview of immune dysregulation and immunomodulatory strategies in endometriosis, a structured literature search was conducted in PubMed/MEDLINE, Embase, Web of Science, and Scopus, with additional searches of ClinicalTrials.gov and the reference lists of relevant publications. The literature search covered studies published from database inception to 22 April 2026. Search terms included combinations of “endometriosis”, “immune microenvironment”, “macrophages”, “NK cells”, “dendritic cells”, “neutrophils”, “mast cells”, “T cells”, “B cells”, “immune checkpoints”, “cytokines”, “fibrosis”, “angiogenesis”, “immune modulation”, and “immunotherapy”. Only English-language publications were included. Priority was given to original studies, clinical trials, systematic reviews and landmark mechanistic investigations relevant to immune cells, cytokines, immune checkpoints, and immunotherapy in endometriosis. The evidence was synthesized qualitatively to summarize current advances in the immunopathogenesis and emerging immunotherapeutic strategies for endometriosis.

## 2. The Immune Microenvironment in Endometriosis

**Figure 2:**
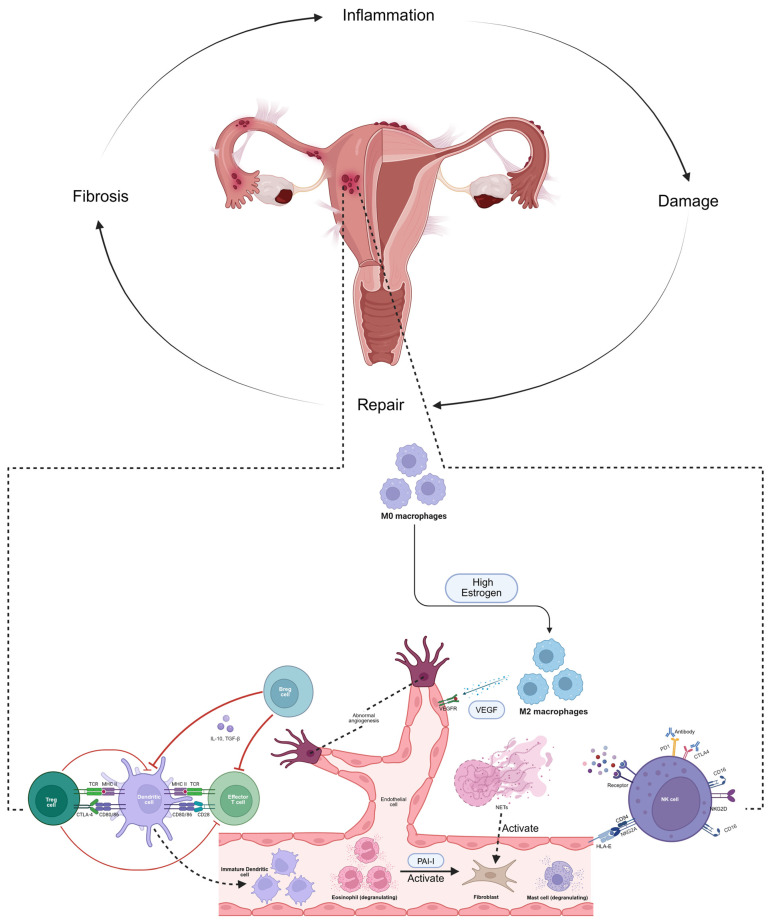
Immune microenvironment driven pathological loop in endometriosis. The schematic illustrates the dynamic interplay between inflammation, tissue damage, repair, and fibrosis in endometriosis. Cyclic progression of inflammation promotes tissue injury, followed by a dysregulated repair process that ultimately contributes to fibrotic remodeling. During this process, macrophages play a central regulatory role. Under high-estrogen conditions, macrophages are polarized toward an M2-like phenotype, which facilitates angiogenesis and lesion progression through the secretion of VEGF. Simultaneously, immune tolerance is established through DCs-mediated activation of Treg cells and suppression of effector T cell responses, partially driven by immunosuppressive cytokines such as IL-10 and TGF-β. NK cell cytotoxicity is impaired, contributing to the immune escape of ectopic endometrial cells. In parallel, activated neutrophils release NETs, and stromal components—including fibroblasts and mast cells—are stimulated via mediators such as PAI-1, promoting extracellular matrix deposition and fibrosis. These coordinated immune and stromal interactions sustain lesion survival, angiogenesis, and chronic inflammation, thereby reinforcing the pathological cycle of endometriosis. Abbreviation: Vascular endothelial growth factor (VEGF); Dendritic Cells (DCs); Interleukin (IL); transforming growth factor-β (TGF-β); neutrophil extracellular traps (NETs); Plasminogen Activator Inhibitor-1 (PAI-1).

Endometriosis is characterized by a cyclic pathological process involving inflammation, tissue damage, aberrant repair, and fibrosis, as illustrated in Figure 2. Persistent inflammation induces repeated tissue injury, followed by dysregulated repair responses that ultimately lead to fibrotic remodeling, thereby reinforcing the inflammatory loop [20,21].

At the center of this process are macrophages. Scavenger receptors are a primary mechanism by which macrophages recognize sloughed endometrial fragments. A primary source of this endometrial debris is retrograde menstruation, which as noted above is a common physiological phenomenon and is generally not associated with disease. Scavenger receptor disfunction disrupts macrophage recognition and removal of endometrial debris and endometrial cells refluxed through the fallopian tubes into the peritoneal cavity during menstruation [22,23,24]. M0 macrophages recruited into ectopic lesions undergo polarization under microenvironmental cues. Elevated local estrogen levels promote macrophage differentiation toward an M2 phenotype, which is associated with immunosuppressive and pro-repair functions [25,26,27]. M2 macrophages secrete pro-angiogenic mediators, particularly VEGF, thereby driving abnormal angiogenesis and supporting lesion survival and expansion [28,29].

DCs further contribute to immune tolerance within the lesion microenvironment. Through antigen presentation via major histocompatibility complex class II (MHC II), DCs preferentially induce Treg cell differentiation while suppressing effector T cell responses. This process is mediated by immunosuppressive cytokines such as interleukin-10 (IL-10) and TGF-β, ultimately promoting immune escape of ectopic endometrial tissue [30,31].

In parallel, NK cell function is impaired in endometriosis. Reduced expression of activating receptors (e.g., Natural Killer Group 2D (NKG2D)) and enhanced inhibitory signaling diminish NK cell cytotoxicity, allowing ectopic cells to evade immune clearance [32,33].

Neutrophils also contribute to disease progression through the release of NETs, which enhance local inflammation and endothelial activation. These processes, together with VEGF signaling, promote aberrant angiogenesis within ectopic lesions [34,35].

At the stromal level, multiple effector cells participate in the repair phase. Fibroblasts are activated and contribute to extracellular matrix deposition, while mast cell degranulation and eosinophil activation amplify inflammatory and fibrotic responses [36,37,38]. Notably, upregulation of PAI-1 inhibits fibrinolysis and promotes extracellular matrix accumulation, thereby driving fibrosis [39,40].

Collectively, these immune and stromal interactions establish a feed-forward pathological loop in which inflammation induces damage, followed by aberrant repair and progressive fibrosis. This integrated network sustains lesion persistence and underlies the chronic progression of endometriosis.

### 2.1. Innate Immune Cells at Endometriotic Lesions

#### 2.1.1. Macrophages

Macrophages are mononuclear phagocytic cells, most of which originate from progenitor cells in the bone marrow [41]. After being released from the circulation, macrophages migrate into tissues where they undergo differentiation in response to local cues, giving rise to distinct subsets depending on the site of pathology and the surrounding microenvironment. Macrophage populations within specific tissues exhibit specialized functions and gene expression profiles, suggesting that macrophages undergo significant alterations within endometriotic lesions [42]. As a key component of the immune system, macrophages exhibit remarkable phenotypic plasticity in response to microenvironmental cues, ranging from strongly pro-inflammatory states involved in pathogen clearance to anti-inflammatory phenotypes associated with tissue protection and repair. To categorize these functional states, numerous studies have classified macrophages into classically activated and alternatively activated phenotypes based on their receptor profiles, secretory patterns, and responses to external stimuli [43]; Classically activated macrophages, referred to as M1 macrophages, are typically induced by interferon-γ (IFN-γ), tumor necrosis factor-α (TNF-α), and lipopolysaccharide (LPS). Accordingly, M1 macrophages produce pro-inflammatory cytokines and chemokines, participating in the early stages of tissue injury, pro-inflammatory responses, and myocyte proliferation. In contrast, alternatively activated macrophages, known as M2 macrophages, are induced by cytokines such as IL-4, IL-10, IL-13, and TGF-β. Upon activation, M2 macrophages secrete anti-inflammatory cytokines, growth factors, and tissue repair mediators, contributing to the resolution phase of inflammation, as well as tissue repair and healing processes [44,45,46].

Studies have demonstrated that, in endometriotic lesions, macrophages exhibit alterations in number, phenotype, and signaling profiles compared with those in normal endometrium. In terms of abundance, Erin Greaves and colleagues provided direct evidence from animal studies showing that bone marrow-derived macrophages accumulate within implanted endometriotic lesions and promote lesion growth, rather than maintaining cyclical homeostasis in accordance with the menstrual cycle [47]. An imbalance in macrophage polarization is also a critical factor in the maintenance of endometriotic lesions. Mei-Fang Nie and colleagues reported that, in the peritoneal fluid of patients with endometriosis, the proportion of CD86^+^ macrophages is decreased, whereas the ratio of CD163^+^/CD86^+^ macrophages is increased, indicating a shift from M1 toward M2 macrophage polarization [48]. In the early stage of the disease, macrophages tend to exhibit an M1 phenotype, secreting high levels of pro-inflammatory cytokines such as IL-1, IL-6, IL-12, IL-23, and TNF-α, thereby sustaining and exacerbating the local inflammatory response. In addition, the aberrant production of chemokines, including IL-8 Chemokine (C-X-C motif) ligand (CXCL8) and members of the CXCL1–17 family, further contributes to the persistence of chronic inflammation [49,50]. In established lesions at later stages, macrophages predominantly adopt an M2 phenotype, secreting anti-inflammatory cytokines such as IL-10 and growth factors including TGF-β, VEGF, and nerve growth factor (NGF), thereby promoting angiogenesis and neurogenesis and contributing to lesion maintenance and stability [46,48,51,52].

Within the local microenvironment of endometriotic lesions, elevated levels of 17β-estradiol are generated due to aromatase-mediated estrogen biosynthesis and the overexpression of estrogen receptor β (ERβ). This hyperestrogenic milieu activates chemokine regulatory pathways in endometriotic cells through estrogen receptor–dependent gene transcription, including C–C motif chemokine ligand 2 (CCL2) Also known as Monocyte Chemoattractant Protein-1 (MCP-1), CCL5, IL-6, IL-8, and CXCL12. These signals establish a chemotactic gradient that promotes the recruitment of circulating monocytes to the lesion site, where they subsequently differentiate into macrophages within the local microenvironment [53,54]. ERβ regulates the production of the CCL2 in endometrial stromal cells via the nuclear factor kappa-B (NF-κB) signaling pathway, thereby promoting the recruitment of macrophages to ectopic lesions and contributing to disease pathogenesis [52]. The local hyperestrogenic milieu also modulates macrophage polarization and function, promoting a pro-repair and pro-angiogenic M2-like phenotype that supports lesion survival and the persistence of chronic inflammation. Collectively, these processes constitute an estrogen–immune–inflammatory crosstalk axis, which represents a key pathological basis underlying the initiation and progression of endometriosis [55].

Endometriosis exhibits several tumor-like features and shares immunopathological similarities with neoplastic diseases, allowing it to be examined from a tumor-like perspective. Tumor-associated macrophages (TAMs) play a pivotal role in the tumor microenvironment and are broadly classified, based on their phenotype and function, into pro-tumorigenic M2 macrophages and anti-tumorigenic M1 macrophages [56]. Studies have shown that M2 macrophages promote tumor cell proliferation, invasion, angiogenesis, and fibrosis, thereby facilitating tumor growth. A similar pattern is observed in endometriosis, where macrophages secrete a variety of factors that support the proliferation of ectopic endometrial tissue, including pro-inflammatory cytokines such as TNF-α, IL-6, and IL-8, as well as growth factors such as VEGF. Under physiological conditions, TNF-α is known to induce apoptosis [57]. However, endometriotic lesion cells exhibit reduced sensitivity to the pro-apoptotic effects of TNF-α, thereby escaping apoptosis and continuing to proliferate at ectopic sites. Van Antwerp and colleagues demonstrated that the TNF-α signaling pathway involves a negative feedback mechanism, in which activation of NF-κB suppresses cell death signaling. Moreover, endometriotic cells can shift TNF-α signaling from the TNFR1-mediated apoptotic pathway toward Tumor Necrosis Factor Receptor 2 (TNFR2)- and NF-κB-driven proliferative pathways, effectively converting TNF-α into a growth-promoting factor for lesion development [58]. The persistent presence of activated macrophages within endometriotic lesions is maintained, at least in part, by their resistance to apoptosis. In the peritoneal environment of endometriosis, sustained activation of the NF-κB signaling pathway leads to upregulation of anti-apoptotic proteins such as B-cell lymphoma-2 (Bcl-2) in macrophages, thereby preventing their cell death and ultimately contributing to disease progression [59]. M2-polarized tumor-associated macrophages exert immunomodulatory effects on other immune cells, including cytotoxic T cells and natural killer cells, thereby attenuating their cytotoxic activity and contributing to the establishment of immune tolerance [60]. Ectopic endometrial tissue can also elicit local immune responses at lesion sites; therefore, evasion of immune surveillance is essential for lesion implantation and growth. The similarities between macrophages in endometriosis and tumor-associated macrophages suggest that M2 macrophage-mediated immunosuppression and immune evasion represent shared features of both endometriosis and cancer. This conceptual overlap provides a potential framework for future research and therapeutic development.

#### 2.1.2. NK Cells

NK cells are key effector cells of the innate immune system, responsible for cytotoxic clearance and immune surveillance through recognition of MHC expression patterns on target cells. As a first line of defense against abnormal and non-self cells, NK cells exert their cytotoxic effects primarily through the release of perforin, granzyme B, and TNF-related apoptosis-inducing ligand (TRAIL). Notably, this cytotoxic capacity is significantly reduced in the peritoneal fluid of patients with endometriosis and is positively correlated with disease severity [61]. NK cell activity is determined by the balance between activating and inhibitory receptor signaling. When MHC I molecules are normally expressed on target cells, inhibitory receptors such as killer cell immunoglobulin-like receptors (KIRs), Killer cell lectin-like receptor D1 (CD94), and Natural Killer Group 2 Member A (NKG2A) engage with these ligands to transmit self-recognition signals, thereby suppressing NK cell cytotoxicity. In contrast, when MHC I expression is absent or downregulated, NK cells detect “missing self” through activating receptors such as NKG2D, Natural Killer cell protein 30 (NKp30), and NKp46, leading to the initiation of cytotoxic responses [62]. NK cells play a critical role in the elimination of abnormal tissues and the maintenance of tissue homeostasis.

In endometriosis, both the number and function of NK cells are markedly altered. Clinical and experimental studies have demonstrated that NK cell cytotoxic activity is reduced in both the peripheral blood and peritoneal fluid of patients with endometriosis [63], the expression of activating receptors, including NKp46 and NKG2D, is decreased, whereas inhibitory receptors such as killer cell immunoglobulin like receptor, two Ig domains and long cytoplasmic tail 1(KIR2DL1), CD94, NKG2A, and programmed cell death protein 1 (PD-1) are significantly upregulated, leading to impaired recognition and clearance of ectopic endometrial cells by NK cells [64]. In addition, high concentrations of immunosuppressive cytokines in the peritoneal fluid, such as TGF-β and IL-10, can further inhibit NK cell activation signaling pathways and downregulate NKG2D expression [65], Moreover, the upregulation of non-classical MHC molecules, including human leucocyte antigen-G (HLA-G) and human leucocyte antigen-E (HLA-E), on the surface of ectopic cells contributes to the establishment of a sustained inhibitory signaling network [66]. These HLA-G and HLA-E molecules interact with inhibitory receptors on NK cells, including killer cell immunoglobulin like receptor, two Ig domains and long cytoplasmic tail 4 (KIR2DL4), CD94, and NKG2A, thereby suppressing cytotoxic responses and ultimately enabling ectopic lesion cells to evade immune clearance.

#### 2.1.3. Dendritic Cells

DCs are professional antigen-presenting cells that serve as a critical link between innate and adaptive immunity. They are broadly classified into conventional dendritic cells (cDCs), also referred to as myeloid dendritic cells (mDCs), and plasmacytoid dendritic cells (pDCs). cDCs drive the activation and differentiation of naïve T cells through efficient antigen uptake, processing, and presentation, accompanied by the upregulation of co-stimulatory molecules such as Cluster of Differentiation 80 (CD80), Cluster of Differentiation 86 (CD86), and MHC class II. In contrast, pDCs are characterized by their robust production of type I interferons and, under specific immunological conditions, can also secrete immunoregulatory cytokines such as IL-10, thereby contributing to the modulation of immune responses [67]. During the maintenance of immune homeostasis in the reproductive tract and peritoneal cavity, mature dendritic cells facilitate the immune recognition and clearance of endometrial debris refluxed into the peritoneal cavity during menstruation through efficient antigen uptake and presentation. In contrast, dysfunction of DCs, particularly a shift toward a tolerogenic phenotype, may impair effective immune clearance and promote local immune tolerance, thereby creating a permissive environment for the establishment and persistence of ectopic lesions [68].

Clinical studies, together with findings from animal models, suggest that within the peritoneal microenvironment of endometriosis, the overall number of DCs may be increased, whereas the proportion of mature DCs is relatively reduced. Flow cytometric analyses indicate that DCs density is elevated in the peritoneal cavity of patients and in early-stage animal models; however, the expression of maturation-associated phenotypes, such as co-stimulatory molecules, is relatively diminished, suggesting a shift of the DCs population toward immature or tolerogenic states. This phenotypic alteration may reflect a remodeling of the local immunoregulatory environment [69]. This phenomenon is considered to be associated with the early establishment phase of endometriotic lesions. Animal model studies further demonstrate that the artificial induction of DCs maturation, for example through LPS stimulation, can inhibit lesion growth to a certain extent, suggesting that the maturation status of DCs plays a regulatory role in disease progression [68]. pDCs are enriched in certain endometriotic lesions and in the peritoneal fluid of patients, where they serve as a major local source of IL-10, thereby promoting angiogenesis and the maintenance of lesions [70]. Clinical and animal data further support a functional contribution of pDCs-derived IL-10 to early lesion angiogenesis, as blockade of IL-10 has been shown to suppress lesion growth [71,72].

In endometriotic lesion tissues and peritoneal fluid, a variety of immunosuppressive factors are present, including IL-10, TGF-β, prostaglandin E_2_ (PGE_2_), and estrogen-related metabolites, which can interfere with the maturation of DCs. This is typically characterized by downregulation of co-stimulatory molecules such as CD80, CD86, and MHC class II, along with reduced IL-12 secretion, thereby impairing DCs-mediated activation of effector T cells and promoting a shift toward a tolerogenic phenotype, which in turn facilitates the differentiation of Treg. In addition, this immunosuppressive microenvironment can induce DCs to upregulate immune checkpoint molecules such as PD-L1 and the metabolic enzyme indoleamine 2,3-dioxygenase (IDO), further enhancing Treg expansion and suppressing effector T cell function through these pathways [71]. Both review and experimental evidence support a central role for IL-10 and TGF-β in inhibiting the maturation of mDCs and promoting the generation of tolerogenic DCs [72].

The work of Suen and colleagues demonstrated that IL-10 secreted by pDCs can stimulate angiogenesis and promote lesion growth during the early stage of disease. Experimental blockade of IL-10 significantly inhibits lesion formation or reduces lesion size, indicating that the pDCs-IL-10-VEGF angiogenic axis represents a functional mechanism underlying early lesion establishment [70].

Recent studies suggest that the expression of local metabolic enzymes can also regulate DCs maturation and function. For example, 11β-hydroxysteroid dehydrogenase type 1 (HSD11B1) is upregulated in endometriotic lesions and can alter local glucocorticoid metabolism, thereby suppressing the maturation and proliferation of mDCs. This provides direct evidence linking microenvironmental metabolism to immune regulation and suggests that targeting metabolic enzymes may represent a potential strategy to restore DCs function [73].

#### 2.1.4. Granulocytes and Mast Cells

The granulocyte family involved in the immune microenvironment of endometriosis primarily includes neutrophils and eosinophils.

Endometrial debris refluxed into the pelvic or peritoneal cavity during menstruation can generate strong chemotactic signals, including CXCL8 (IL-8), CXCL1, and granulocyte colony-stimulating factor (G-CSF), thereby inducing rapid and substantial infiltration of neutrophils [74]. In the early stage, neutrophils participate in the clearance of tissue debris; however, when clearance is dysregulated or stimuli persist, they can disrupt the local basement membrane through the release of enzymatic and oxidative mediators, including matrix metalloproteinases (MMPs), neutrophil elastase (NE), and reactive oxygen species (ROS), thereby facilitating the adhesion and implantation of ectopic endometrial tissue. This process has been consistently highlighted in recent experimental studies and reviews [75]. Studies have shown that levels of NETs are elevated in endometriotic lesions and in the peritoneal fluid of patients. NETs, composed of extracellular DNA, histones, and enzymes such as neutrophil elastase, can function as adhesive and pro-fibrotic scaffolds. These structures facilitate the adhesion of ectopic endometrial cells, promote the recruitment of local inflammatory cells, and activate fibroblasts as well as epithelial–mesenchymal transition (EMT) pathways, thereby driving lesion expansion and fibrosis [76,77]. Studies have demonstrated the presence of NETs in endometriosis and provided evidence of their association with disease severity. Moreover, inhibition of NETs in mouse models has been shown to attenuate lesion progression [76]. NETs and damage-associated molecular patterns (DAMPs) can activate the NF-κB signaling pathway via Toll-like receptors Toll-like receptor 4 (TLR4) and Toll-like receptor 2 (TLR2), thereby inducing the expression of pro-inflammatory cytokines such as IL-1β, TNF, and IL-6 and establishing a positive feedback inflammatory loop [78]. Activation of neutrophils and other immune cells further promotes NLRP3-mediated IL-1β release, synergistically driving local inflammation and pain sensitization [78]. In addition, ROS and MMPs contribute to extracellular matrix degradation and facilitate the invasion of endometrial fragments [79].

Although studies on eosinophils in endometriosis remain limited, recent pathological investigations have reported their enrichment in certain lesions, particularly in fibrotic or chronic lesion regions, often accompanied by evidence of degranulation [38]. Eosinophils can secrete mediators such as PAI-1, TGF-β, and major basic protein (MBP), which promote fibroblast activation and collagen deposition, thereby contributing to lesion-associated fibrosis. Evidence from both animal studies and human pathological samples supports the hypothesis that eosinophils play a role in fibrotic processes [80].

Histological, transcriptomic, and proteomic analyses have demonstrated that mast cells are increased in number and exhibit enhanced degranulation within endometriotic lesions. The concentrations of their degranulation products, including histamine, tryptase, chymase, TNF, IL-6, IL-8, and prostaglandin D_2_ (PGD_2_), are elevated in the local lesion microenvironment. These mediators can directly induce vasodilation, increase vascular permeability, and promote extracellular matrix degradation and remodeling. In addition, their close spatial association with nerve endings contributes to pain sensitization and the development of chronic pain [81,82].

IL-33, acting as an alarmin, is upregulated in endometriosis and can directly activate mast cells via its receptor ST2 also known as interleukin 1 receptor-like 1 (IL-1RL1), thereby inducing the production of a wide range of inflammatory mediators as well as pro-fibrotic and pro-angiogenic factors, including VEGF, MMPs, and IL-6 [83]. In addition, IL-33 can activate group 2 innate lymphoid cells (ILC2s) and promote Th2-type immune responses, thereby indirectly enhancing eosinophil- and mast cell-associated immune activities [83]. Recent studies indicate that IL-33/ST2 signaling is functionally associated with EMT, angiogenesis, and pain-related pathways, including the janus kinase Signal Transducer and Activator of Transcription 3 (JAK–STAT) signaling cascade, in endometriotic lesions [82].

In endometriotic lesions, infiltrating mast cells are significantly increased in number and exhibit a highly activated state. These cells release a wide range of bioactive mediators through degranulation, thereby exerting broad effects on the lesion microenvironment [37,84]. On the one hand, mast cells contain abundant serine proteases, such as tryptase and chymase, which can degrade the extracellular matrix and induce neighboring cells to release MMPs, thereby promoting matrix remodeling and structural alterations of lesion tissues [37,81]. On the other hand, these cells secrete a wide range of inflammatory cytokines and chemokines, including IL-6, IL-8, CXCL8, and TNF-α, as well as pro-angiogenic factors such as VEGF. The latter stimulates endothelial cell proliferation and neovascularization, providing nutritional support for ectopic endometrial tissue and facilitating lesion growth [37]. In addition, mast cells are closely associated with nerve fibers within lesions, and their released mediators are considered to contribute to pain sensitization mechanisms. Collectively, these mediators act synergistically in chronic inflammation, angiogenesis, and tissue remodeling, thereby establishing an immunoinflammatory microenvironment that promotes disease progression.

### 2.2. Adaptive Immune Cells at Endometriotic Lesions

#### 2.2.1. T Cells

T cells are central regulators of the immune microenvironment in endometriosis. A distinct remodeling of T cell lineages is observed in lesions and peritoneal fluid, characterized by an increase in pro-inflammatory effector subsets, including Cluster of Differentiation 4 (CD4^+^) T helper cell 17 (Th17) and, to a lesser extent, Th1 phenotypes, alongside the expansion of immunosuppressive regulatory T cells (Treg) and upregulation of immune checkpoint molecules [85]. Together, these changes establish a microenvironment in which inflammation and immune tolerance coexist, thereby supporting the adhesion and growth of ectopic endometrial tissue while suppressing effective immune clearance of lesions [86].

Clinical and experimental studies have shown that patients with endometriosis frequently exhibit increased proportions of Th17 cells and elevated IL-17 levels in peripheral blood, peritoneal fluid, and lesion tissues, whereas the proportion of Treg is relatively decreased or functionally reprogrammed compared with controls [85]; A shift toward a pro-inflammatory Th17 phenotype promotes inflammation and angiogenesis through the induction of cytokines such as IL-6, IL-8, and VEGF, while Treg-derived IL-10 and TGF-β contribute to local immunosuppression and support lesion persistence. This balance is regulated by a cytokine network involving IL-1β, IL-6, TGF-β, and IL-23, with the IL-23/Th17 axis being highlighted in multiple studies as a key contributor to lesion maintenance and its association with infertility [87].

In the healthy uterine environment, CD8^+^ T cells are responsible for the clearance of abnormal cells. However, in endometriosis, CD8^+^ T cells within lesions frequently exhibit a functionally impaired or exhaustion-like phenotype, characterized by the upregulation of inhibitory molecules such as PD-1, Lymphocyte activation gene 3 (LAG-3), and T cell immunoglobulin and mucin-domain containing protein 3 (TIM-3). This is accompanied by reduced production of effector molecules, including IFN-γ, perforin, and granzyme B, ultimately leading to diminished cytotoxic clearance of ectopic endometrial cells [88]. This exhausted and inhibitory phenotype is associated with persistent local antigenic stimulation, elevated levels of immunosuppressive cytokines such as TGF-β and IL-10, and increased expression of immune checkpoint ligands, including Programmed Cell Death Ligand 1 (PD-L1) and HLA-G [89]. Studies and reviews have consistently reported the involvement of immune checkpoint molecules, including PD-1, PD-L1, Cytotoxic T-Lymphocyte Antigen 4 (CTLA-4), and soluble checkpoint factors, in the pathophysiology of endometriosis, with associations to infertility and disease severity. These pathways contribute to immune evasion by suppressing T cell activation and enhancing Treg function. Based on these observations, immune checkpoint signaling has emerged as a potential therapeutic target [89,90]. Pro-inflammatory cytokines, including IL-17 (derived from Th17 cells), IL-1β, IL-6, and TNF-α, can amplify the inflammatory cascade within lesions via activation of the NF-κB and mitogen-activated protein kinase (MAPK) signaling pathways, thereby inducing the expression of MMPs and VEGF, which promote extracellular matrix degradation, adhesion, and angiogenesis [13,86]. In contrast, Treg-derived cytokines such as IL-10 and TGF-β suppress effector T cell responses through STAT3 and Smad signaling pathways and induce a tolerogenic phenotype. These factors can also reduce the cytotoxic activity of NK cells and CD8^+^ T cells, acting in concert with the local upregulation of HLA-G and HLA-E [55,86].

#### 2.2.2. B Cells

B cells in endometriosis exhibit a dual role characterized by the coexistence of pro-inflammatory humoral immunity and localized immune tolerance. Histological and transcriptomic evidence indicates that ectopic lesions are frequently enriched with plasma cells and accompanied by local immunoglobulin (IgM/IgG) deposition, suggesting active humoral immune responses within the lesions. Meanwhile, macrophages and stromal cells within lesions can highly express B-cell activating factor (BAFF, also known as BLyS), which, through signaling via BAFF-R, transmembrane activator and calcium modulator and cyclophilin ligand interactor (TACI), and B-cell maturation antigen (BCMA), supports B cell survival, plasma cell differentiation, and local antibody production. This process contributes to the maintenance of a persistent, antibody-mediated inflammatory state within the lesion microenvironment [91,92].

Clinical studies of serum and peritoneal fluid further demonstrate that patients with endometriosis frequently exhibit distinct profiles of auto-antibodies targeting endometrial-related antigens, such as α-enolase and tropomyosin, as well as hormone-related antigens. These auto-antibodies and immune complexes can activate the complement system and drive inflammatory responses in macrophages and neutrophils via Fc receptor engagement, thereby promoting extracellular matrix degradation, adhesion formation, and fibrosis, and potentially impairing reproductive function [92,93].

On the other hand, regulatory B cells (Breg) exert their functions primarily through IL-10, TGF-β, and PD-L1. Within the immune microenvironment of endometriosis, Breg cells can suppress the activity of DCs, effector T cells, and natural killer cells, thereby establishing a degree of local immune tolerance that facilitates immune evasion of ectopic tissues. The balance between Breg and pro-inflammatory B cell subsets, as well as their functional states, may determine the shift of lesions toward either an inflammation-dominant or tolerance-dominant phenotype [92,94].

At the mechanistic level, B cell receptor (BCR) activation, together with TLR-mediated polyfunctional stimulation—such as via TLR9—and downstream transcriptional networks including NF-κB, Phosphatidylinositol 3-Kinase/Protein Kinase B Signaling Pathway (PI3K–AKT), and STAT3, collectively drive B cell activation, proliferation, and differentiation into plasma cells. Concurrently, BAFF signaling forms a positive feedback loop with local inflammatory cytokines such as IL-6 and TNF-α, thereby enhancing humoral immune output and promoting lesion stabilization [91,95]. Genetic evidence from Mendelian randomization studies further suggests a causal association between B cell proportions, related immune phenotypes, and the risk of endometriosis, supporting a functional role for B cells in the disease pathogenesis [94]. Notably, recent advanced genetic epidemiological evidence from multi-phenotype Mendelian randomization (MR) studies has provided a causal foundation for these cellular observations. Beyond establishing causal associations between B cell traits and endometriosis risk, genomic causal inference has mapped an intricate network involving UDP-Glucose Ceramide Glucosyltransferase (UGCG) inhibition, lipid metabolism, and systemic inflammatory proteins. These MR findings demonstrate that inherited genetic variants regulating immune cell proportions and cytokine cascades hold a direct causal relationship with disease susceptibility, thereby shifting the paradigm from observational correlations to genetically validated therapeutic targets [96,97].

In summary, B cells exert dual functions in endometriosis by amplifying local inflammation and tissue remodeling through antibody production and immune complex formation, while also promoting local immune tolerance via regulatory mechanisms. Therapeutic strategies based on two principal axes—BAFF-driven plasma cell maintenance and Breg-mediated immunosuppression—may represent promising directions for future treatment and biomarker development.

## 3. Immune Cells Therapies for Endometriosis

Recent studies have increasingly revealed that endometriosis is not only an estrogen-dependent disease but also a chronic inflammatory pathological condition driven by immune dysregulation [98]. The endometriotic microenvironment (EME) is enriched with diverse immune cell populations, including macrophages, T cells, B cells, NK cells, and DCs, which under the regulation of cytokine networks such as IL-6, IL-10, TNF-α, and TGF-β, collectively determine lesion survival, angiogenesis, and immune evasion [99]. Similar to the tumor microenvironment, the bidirectional imbalance of immune cell functions in the EME—characterized by the coexistence of pro-inflammatory activity and immunosuppression—provides a rationale for immunotherapeutic intervention [100]. Current research focuses on modulating the activity of specific immune cell subsets, blocking key cytokine signaling pathways such as IL-6 and targeting immune checkpoints including PD-1 and PD-L1 to restore local immune homeostasis [101].

Although immunotherapy for endometriosis remains at an experimental stage, evidence from animal models and early in vitro studies indicates its potential to reduce inflammation, inhibit lesion growth, and alleviate pain [102]. In the subsequent sections of this review, we systematically discuss the clinical studies and experimental immunotherapeutic strategies targeting immune cells in endometriosis.

To facilitate cross-comparison among different immune cell-targeted therapeutic strategies, Table 1 summarizes the major immune cell subsets involved in endometriosis, their representative targets and signaling pathways, corresponding therapeutic approaches, current stages of translational development, and major safety concerns. Despite substantial mechanistic advances, most immunotherapeutic strategies for endometriosis remain at the preclinical or early exploratory stage, with limited clinical validation and unresolved reproductive safety issues.

### 3.1. Targeted Macrophage Therapy for Endometriosis and Recent Advances in Research

Macrophages are recruited to endometriotic lesions and the peritoneal cavity, where they predominantly exhibit a pro-pathological M2-like immunoregulatory phenotype. These cells contribute to the maintenance of inflammation, angiogenesis, extracellular matrix remodeling, and neuro-sensitization, thereby supporting lesion survival and growth. Clinical and animal studies have reported upregulation of colony-stimulating factor-1 (CSF-1) and its receptor CSF-1R in endometriotic lesions, which is associated with macrophage recruitment and phenotypic polarization [112]. Given the high plasticity of macrophages, therapeutic strategies may involve either inhibiting their recruitment or depleting pro-pathogenic macrophages within lesions, as well as reprogramming them toward a phagocytic, M1-like phenotype to reshape the lesion microenvironment. Both review and experimental studies support this bidirectional intervention approach [113].

Based on current evidence, targeting strategies can be broadly categorized into inhibition of recruitment, suppression of survival, macrophage depletion, phenotypic reprogramming, gene therapy and cell transplantation, as well as indirect approaches.

Inhibition of recruitment: targeting chemotactic and mobilization pathways. By blocking the recruitment of macrophages from the bone marrow to lesions, the number of lesion-associated macrophages can be reduced. Animal studies have demonstrated that the CCL2–CCR2 axis plays a critical role in macrophage recruitment and lesion-associated angiogenesis; genetic knockout or antibody-mediated blockade of this pathway can alter macrophage infiltration and affect lesion development [52,114].

Suppression of survival: targeting the CSF-1/CSF-1R axis. CSF-1, also known as macrophage colony-stimulating factor (M-CSF), is a critical regulator of macrophage biology. Increased expression of CSF-1 and CSF-1R has been demonstrated in endometriotic lesions, supporting the involvement of the CSF-1/CSF-1R signaling axis in the pathogenesis of endometriosis [112]. A recent study further demonstrated that the dual CSF-1R/KIT inhibitor pexidartinib suppresses inflammatory signaling and reduces the viability of endometriotic cells in vitro, providing preliminary evidence supporting further evaluation of CSF-1R-targeted therapeutic strategies in endometriosis [115].

Macrophage depletion: local delivery strategies, including encapsulated small molecules and liposomal formulations (e.g., clodronate-loaded liposomes), as well as systemic CSF-1R inhibition, can reduce macrophage burden [115]. In murine studies, antibody-mediated depletion of macrophages has been shown to alter disease progression [116]; however, potential adverse effects, including systemic immunodeficiency and impaired wound healing, should be carefully considered.

Reprogramming (re-polarization): shifting M2-like macrophages toward an M1 phenotype to restore phagocytic and clearance functions. Among various macrophage-reprogramming strategies, PI3Kγ inhibition—particularly via the CD16–PI3Kγ axis—has been explored to shift macrophages from an M2-dominant state to an M1-like state, thereby restoring phagocytic activity and pro-inflammatory functions in the endometriotic microenvironment [29,117]. Such strategies have shown progress in studies of tumor-associated macrophages and may be translatable to endometriosis. Emerging evidence indicates that endometriosis-derived exosomes and microRNAs, such as miR-301a-3p, can drive macrophage polarization toward the M2 phenotype via the PTEN–PI3K signaling pathway. Inhibition of PI3K signaling or targeting exosome-mediated communication has been shown to reverse this polarization, suggesting that the PI3K pathway represents a potential therapeutic target. Furthermore, in oncology, inhibition of PI3Kγ has been demonstrated to suppress immunosuppressive myeloid cells and enhance the efficacy of immunotherapy. Collectively, these findings may inform future therapeutic strategies for endometriosis [104,118].

Gene therapy and cell transplantation: adoptive transfer of ex vivo–polarized M1 macrophages or engineered macrophages into the peritoneal cavity has been proposed to enhance the clearance of ectopic endometrial tissue. In addition, macrophages can be utilized as carriers for targeted delivery of therapeutic agents or genes, enabling the localized release of anti-inflammatory and pro-clearance molecules. Recent studies have explored the feasibility of M1 macrophage-based cell therapy in animal models of endometriosis, demonstrating potential therapeutic efficacy. However, these approaches remain at an early exploratory stage and require rigorous evaluation of reproductive safety and inflammation-related adverse effects [119].

Indirect strategies: targeting upstream signals that regulate macrophage function, including cytokines and metabolic pathways. Inhibition of key lesion-associated factors such as IL-6, STAT3, TGF-β, and VEGF can reduce macrophage recruitment and immunosuppressive activity, while simultaneously suppressing angiogenesis and fibrosis. IL-6 blockade, in particular, has been proposed in theoretical frameworks and early study concepts for the treatment of endometriosis [116,120,121,122].

Current clinical and preclinical evidence: the findings based on a search of ClinicalTrials.gov for studies related to macrophages and endometriosis are summarized in Table 2. At present, the number of registered clinical trials explicitly focusing on macrophages in endometriosis remains limited, with approximately half being observational studies and the other half interventional in design. These studies are primarily centered on immune cell functional assessment, inflammatory signaling, and correlations with pathological features. Notably, there are currently no registered interventional clinical trials specifically targeting macrophages with therapeutic agents. Although preclinical evidence—including cell-based models and tissue expression profiling—has demonstrated mechanistic roles for the CSF-1/CSF-1R axis, inflammatory cytokines, and macrophage polarization in the pathogenesis of endometriosis, no interventional trials targeting these pathways (e.g., CSF-1R inhibitors or anti–IL-6 therapies) have been registered for endometriosis in clinical trial databases to date. This gap highlights that the translational development of immunotherapy for endometriosis remains at an early stage, with current efforts largely focused on mechanistic investigations and biomarker discovery—such as macrophage phenotypes and inflammatory microenvironment signatures—rather than on therapeutic intervention studies.

### 3.2. Targeted NK Cells Therapy for Endometriosis and Recent Advances in Research

Studies have demonstrated that NK cells in patients with endometriosis exhibit markedly reduced cytotoxicity, upregulated inhibitory signaling, and functional impairment. These dysfunctional NK cells facilitate immune evasion of ectopic endometrial cells, thereby promoting lesion survival. In particular, increased expression of inhibitory receptors on NK cells—such as KIR2DL1, NKG2A, leukocyte immunoglobulin-like receptor B1 (LILRB1), and leukocyte immunoglobulin-like receptor B2 (LILRB2)—and their interaction with corresponding ligands, including HLA-E and HLA-G, significantly suppress cytotoxic activity and represent a key mechanism underlying NK cells dysfunction [127,128].

In addition, the inflammatory microenvironment surrounding lesions—characterized by elevated levels of TGF-β, metabolic intermediates such as L-kynurenine, and immunosuppressive cytokines—can further exacerbate NK cells dysfunction by altering their metabolic state and intracellular signaling pathways. This, in turn, weakens the immune clearance of ectopic endometrial tissues [129].

Although immunotherapeutic strategies targeting natural killer cells in endometriosis remain at an early exploratory stage, several mechanistic approaches have been proposed for translational investigation. In principle, blockade of inhibitory receptors expressed on NK cells—such as KIR2DL1, CD94, NKG2A, and LILRB—and disruption of their interactions with corresponding ligands may restore NK cells cytotoxic activity. This strategy has been explored in the context of tumor immunotherapy and has been proposed as a potential direction for immune modulation in endometriosis [65,104]. In vitro studies and animal models have demonstrated that cytokines such as IL-2 and IL-15 can enhance NK cells activity and may improve their capacity to eliminate ectopic endometrial cells. However, the potential risks of adverse effects and immune dysregulation require further comprehensive evaluation [105]. Reports indicate that a pilot study conducted in China has explored the therapeutic potential of autologous natural killer cell-based therapy in endometriosis. In this trial, NK cells therapy was administered in combination with conventional hormonal treatment using a gonadotropin-releasing hormone (GnRH) agonist. The study evaluated outcomes including safety, endocrine parameters, pain scores, and pregnancy-related endpoints, representing an initial clinical attempt to assess autologous NK cells therapy in humans with endometriosis. This information is derived from the registered clinical trial summary (NCT03948828) and related review literature [104].

To date, among international clinical trial registries, including ClinicalTrials.gov, only one study has been registered that is directly related to NK cell-based immunotherapy in endometriosis: NCT03948828 (Clinical Study of NK Cells in the Treatment of Severe Endometriosis). This trial investigates the safety and efficacy of NK cell therapy in combination with standard treatment, and clinical data collection is currently ongoing. While this study represents an initial clinical exploration of NK cell-based therapy, no results have yet been publicly reported. Furthermore, most existing studies remain at the preclinical or mechanistic validation stage, and large-scale, multicenter clinical evidence is still lacking.

Current NK cell-based therapeutic strategies for endometriosis face several major challenges. These include concerns regarding immune safety and potential reproductive risks, as NK cells play critical roles in normal pregnancy, and excessive activation may impair endometrial receptivity. In addition, issues of target specificity and immune tolerance remain unresolved, particularly how to enhance the selective cytotoxicity of NK cells against ectopic endometrial tissue without inducing systemic autoimmunity. Furthermore, clinical evidence remains limited, with existing studies characterized by small sample sizes and heterogeneous designs, precluding the establishment of standardized therapeutic approaches [101]. Future research should therefore focus on strategies such as inhibitory receptor blockade, cytokine-mediated activation, NK cells genetic engineering, and combinational immunomodulatory approaches. In parallel, well-designed multicenter, randomized controlled trials are urgently needed to validate therapeutic efficacy and comprehensively assess safety.

### 3.3. Targeted Dendritic Cells Therapy for Endometriosis and Recent Research Advances

DCs serve as a critical bridge between innate and adaptive immunity. Mature DCs efficiently present antigens and activate effector T cells, whereas immature or tolerogenic DCs, as well as pDCs, can secrete immunosuppressive cytokines such as IL-10, thereby promoting Treg differentiation and suppressing cytotoxic immune responses. Studies have shown that both the number and phenotype of DCs are altered in endometriotic lesions and peritoneal fluid, including increased recruitment of pDCs and impaired mDCs. Moreover, animal model data indicate that pDCs-derived IL-10 can promote early lesion angiogenesis and tissue growth. From a mechanistic perspective, strategies aimed at correcting the tolerogenic state of DCs or blocking pDCs-derived IL-10 signaling may represent potential approaches for modulating the immune microenvironment in endometriosis [130].

Suen et al. demonstrated in animal models that blockade of IL-10 reduces lesion size and suppresses angiogenesis, indicating that this axis represents a priority target for validation [70]. In the context of pDCs-mediated immunosuppression, local administration of anti-IL-10 neutralizing antibodies can attenuate immunosuppressive signaling. Meanwhile, antibodies targeting Blood Dendritic Cell Antigen 2 (BDCA2) may inhibit the activation and recruitment of pDCs. In addition, oligonucleotide-based inhibitors directed against pDCs-specific signaling pathways have emerged as potential therapeutic strategies to further modulate the immunological functions of pDCs [107,131]. This approach may offer new insights into the treatment of endometriosis.

In oncology, DC vaccines have been demonstrated to induce antigen-specific immune responses. In the context of endometriosis, reviews on DCs dysfunction suggest that restoration of DCs maturation represents a theoretically feasible approach; however, its potential impact on endometrial receptivity warrants careful evaluation. DCs activity may be enhanced through local or systemic administration of TLR agonists, such as CpG-ODN, in combination with factors that promote DCs expansion, including GM-CSF or FLT3L. Furthermore, short-term administration of immunostimulatory cytokines, such as IL-12 and IL-15, may further enhance antigen presentation and promote a Th1-skewed immune response. Additionally, lesion-derived antigens may be loaded onto ex vivo-matured autologous DCs followed by reinfusion, thereby eliciting antigen-specific immune responses [71].

Targeted inhibition of immunoregulatory pathways, such as IDO and STAT3, or metabolic reprogramming strategies—including suppression of fatty acid oxidation and modulation of mammalian target of rapamycin (mTOR) signaling—may promote the transition of DCs from a tolerogenic state toward a pro-inflammatory phenotype with enhanced antigen-clearing capacity. From a therapeutic perspective, IDO inhibitors, STAT3 small-molecule inhibitors, or metabolic modulators may be employed, with a preference for local delivery in combination with short-term immunostimulatory interventions to minimize the risk of systemic toxicity. Accumulating evidence from reviews and preclinical studies indicates that these signaling pathways play pivotal roles in the establishment and maintenance of DCs-mediated immune tolerance. Furthermore, related targeting strategies have already gained preliminary clinical experience in the field of cancer immunotherapy, providing a translational rationale for their potential application in other diseases [71].

In the treatment of peritoneal inflammatory diseases and cancer, local delivery strategies have accumulated a certain degree of safety data. In endometriosis, local therapy is also clinically feasible, as drug or cell-based formulations can be administered during laparoscopic procedures. Specific approaches include the use of nanocarriers or liposomal delivery systems, biodegradable microspheres, and intraperitoneal sustained-release devices to achieve controlled drug release. In addition, ex vivo-matured or engineered cells, such as dendritic cells, may be directly injected into the peritoneal cavity via laparoscopy. Localized delivery of drugs, antibodies, or oligonucleotide-based agents to the peritoneal cavity or perilesional regions may increase local therapeutic concentrations and enhance efficacy, while reducing systemic exposure and thereby minimizing potential effects on overall endometrial physiological function [71].

To date, no registered interventional studies on ClinicalTrials.gov have specifically focused on DC-targeted therapies for endometriosis. Along the translational pathway from exploratory studies to clinical application, a critical initial step is the systematic characterization of immune cell lineages and their functional states in large-scale human clinical samples. This can be achieved by integrating single-cell RNA sequencing (scRNA-seq) with immunohistochemistry (IHC) to perform comprehensive phenotypic and functional analyses of DCs subsets in lesion tissues and peritoneal fluid. Particular emphasis should be placed on evaluating the abundance and activation status of pDCs, cDCs and mDCs. Concurrently, the expression profiles of immunosuppressive molecules, including IL-10, IDO, and PD-L1, should be assessed to systematically identify key immunoregulatory pathways and prioritize potential therapeutic targets. For instance, the IL-10 signaling axis derived from pDCs or IDO-mediated immune tolerance pathways may represent promising targets, thereby providing a foundation for subsequent mechanistic studies and therapeutic strategy development [69,132].

In the preclinical stage, immunomodulatory strategies should be evaluated in humanized or conventional murine models of endometriosis. Representative approaches include anti-IL-10 neutralizing antibodies, TLR agonists (e.g., Cytosine–Phosphodiester–Guanine (CpG)), or combinatorial regimens such as FMS-like tyrosine kinase 3 ligand (FLT3L) with TLR agonists. Therapeutic efficacy should be assessed by measuring lesion volume, angiogenesis, T cell and NK cell activity, as well as remodeling of the local immune microenvironment. Importantly, endometrial receptivity and pregnancy outcomes should also be evaluated to comprehensively determine both therapeutic efficacy and reproductive safety [70,72].

At the stage of clinical translation, small-scale exploratory studies are recommended, preferably using randomized controlled designs with placebo or standard-of-care treatments as comparators. Local administration strategies should be prioritized to enhance site-specific drug accumulation while minimizing systemic adverse effects. Primary endpoints may include pain scores (e.g., VAS or NRS), imaging-based assessment of lesion size, and dynamic changes in local immune biomarkers. In addition, reproductive endocrine parameters, including hormone levels, menstrual cycle characteristics, and pregnancy rates, should be systematically monitored to evaluate both therapeutic efficacy and potential impacts on reproductive function [71].

Safety considerations are critical in the development of immunomodulatory therapies. Given the essential role of DCs in endometrial immune tolerance and embryo implantation, modulation of DCs function may influence endometrial receptivity and pregnancy outcomes; therefore, pregnancy-related endpoints should be incorporated into long-term follow-up. Moreover, excessive activation of DCs or strong pro-inflammatory stimulation may induce off-target inflammation or increase the risk of autoimmunity. Thus, dosing regimens should be carefully optimized, and localized delivery approaches are preferred. Finally, considering the marked heterogeneity of endometriosis in terms of lesion subtype, menstrual cycle phase, and prior treatment history, appropriate patient stratification is necessary to improve interpretability and enable precision intervention [132].

### 3.4. Research Progress in Targeted Therapies for Endometriosis Targeting Granulocytes and Mast Cells

Neutrophils and their derived NETs have recently emerged as important contributors to the pathogenesis of endometriosis. Elevated levels of NETs have been detected in peritoneal fluid and lesion tissues, where they are associated with inflammation, cellular adhesion, and fibrosis. Mechanistically, NETs form DNA–protein meshworks enriched in granular enzymes, which promote cell adhesion, extracellular matrix remodeling, and amplification of local inflammatory responses, thereby facilitating lesion establishment and persistence [77,78].

Neutrophil recruitment is primarily regulated by the IL-8/CXCL8–CXCR1/2 chemokine axis, which plays a critical role in early inflammatory responses and angiogenesis. Experimental studies have demonstrated that inhibition of CXCR2 signaling reduces neutrophil infiltration and suppresses early lesion formation, highlighting this pathway as a potential therapeutic target [79,133]. In addition, direct targeting of NETs represents a promising strategy. Approaches such as Deoxyribonuclease I (DNase)-mediated degradation of NET structures, inhibition of peptidylarginine deiminase 4 (PAD4), or suppression of neutrophil elastase activity may effectively block NET formation. However, current evidence is largely derived from preclinical studies or other inflammatory disease models, and clinical validation in endometriosis remains limited [35,78].

Eosinophils have been observed to infiltrate certain endometriotic lesions and may contribute to fibrosis through the secretion of profibrotic mediators such as TGF-β and PAI-1. Nevertheless, their functional role in disease progression remains poorly defined. Given the clinical success of IL-5/IL-5R-targeted therapies (e.g., mepolizumab, reslizumab, and benralizumab) in eosinophil-associated disorders, such strategies may hold exploratory potential in selected patient subsets, although further validation is required [38,80,134].

Mast cells are significantly increased in endometriotic lesions and are often found in an activated or degranulated state. Activated mast cells release a variety of mediators, including tryptase, chymase, histamine, and pro-inflammatory cytokines (e.g., IL-6, IL-8, and VEGF), thereby contributing to angiogenesis, extracellular matrix remodeling, and pain sensitization. The IL-33/ST2 signaling axis is recognized as a key pathway in mast cell activation, promoting inflammation, EMT and fibrosis [37,83]. Accordingly, mast cell stabilizers (e.g., cromolyn sodium and ketotifen) and blockade of the IL-33/ST2 axis have been proposed as potential therapeutic strategies; however, robust clinical evidence is still lacking [111].

From a translational perspective, targeting innate immune cells presents several challenges. To date, there is a lack of registered clinical trials specifically targeting granulocytes or mast cells in endometriosis, and most evidence remains at the preclinical stage. Importantly, neutrophils and mast cells play essential physiological roles in endometrial repair and embryo implantation. Therefore, their systemic inhibition or excessive activation may adversely affect endometrial receptivity and pregnancy outcomes, underscoring the need to incorporate reproductive safety endpoints in early-phase clinical studies [111]. Given the heterogeneity of endometriosis, therapeutic benefits may be restricted to patient subsets characterized by specific immune signatures, such as elevated NETs or activation of the IL-33/ST2 axis. Biomarker-driven stratification strategies—such as assessing NET-associated markers, IL-8 levels, IL-33/ST2 signaling components, and tryptase expression—may improve patient selection and therapeutic precision. Furthermore, considering the potential risks of systemic immune modulation, including increased susceptibility to infection and impaired tissue repair, localized delivery systems or short-term intervention regimens may offer a safer and more effective approach [37,78].

### 3.5. Research Progress in Targeted T Cells Therapies for Endometriosis

Significant T cell remodeling has been observed in the immune microenvironment of endometriotic lesions. Both CD4^+^ and CD8^+^ T cells exhibit alterations in distribution, phenotype, and function, including features of T cell exhaustion, upregulation of inhibitory receptors, and an imbalance between Th17 and Treg cells. These changes are closely associated with disease progression, angiogenesis, pain, and infertility. Notably, although CD8^+^ T cells are often enriched within lesions, their functional heterogeneity and impaired cytotoxicity suggest that T cell dysfunction may represent a key mechanism underlying lesion persistence [135]. Multiple T cell-related regulatory pathways are activated in endometriotic lesions, including immune checkpoints (PD-1/PD-L1 and CTLA-4) as well as signaling cascades such as TGF-β/Smad, STAT3, and retinoic acid-related orphan receptor γt (RORγt). These pathways collectively contribute to immunosuppression, chronic inflammation, and altered T cell differentiation. While immune checkpoint blockade has achieved substantial clinical success in oncology, its application in endometriosis remains largely exploratory, with evidence primarily derived from mechanistic studies, in vitro experiments, and limited preclinical data. These pathways nevertheless provide a strong theoretical basis for T cell-targeted therapeutic interventions [71,136].

Immune checkpoint modulation represents a potential strategy to restore T cell function. Blockade of PD-1, PD-L1, or CTLA-4 perhaps alleviate inhibitory signaling, thereby partially rescuing CD8^+^ T cell cytotoxicity and enhancing clearance of ectopic endometrial cells. However, given the physiological role of immune tolerance in endometrial receptivity and embryo implantation, the safety and feasibility of checkpoint inhibition in endometriosis require careful evaluation, particularly with respect to reproductive outcomes [13,137].

Therapeutic targeting of Treg cells is highly context-dependent. In immunosuppressive microenvironments dominated by excessive Treg activity, selective inhibition of Tregs may help restore effector T cell responses. Conversely, in settings characterized by excessive inflammation and impaired Treg function, Treg supplementation or adoptive transfer may suppress pathological inflammation and slow disease progression. Preclinical studies in murine models have shown that systemic or local Treg transfer can reduce inflammatory responses and inhibit lesion growth; however, clinical validation is still lacking [109].

Adoptive T cell therapies, including chimeric antigen receptor T cells (CAR-T), T cell receptor-engineered T cells (TCR-T), and other engineered T cell approaches, represent a theoretically attractive strategy for targeted elimination of ectopic endometrial cells. These approaches perhaps rely on the identification of lesion-specific or overexpressed antigens to enable selective targeting. However, several challenges hinder their translational application. These include the lack of highly specific target antigens with minimal expression in normal tissues, limited T cell infiltration into ectopic lesions, the presence of an immunosuppressive microenvironment, and T cell exhaustion. In addition, the potential impact on normal endometrial physiology and reproductive function must be carefully considered. To date, no registered clinical trials have specifically evaluated CAR-T or TCR-T therapies in endometriosis, and current clinical development remains largely confined to oncology [138,139].

Epigenetic and metabolic interventions provide additional avenues to restore T cell function. Modulation of signaling pathways such as STAT3, as well as correction of local immunosuppressive conditions induced by hypoxia and lactate accumulation, may reprogram T cell activity and rebalance Th17/Treg dynamics. Although these strategies have been proposed in recent reviews, they remain at an early, predominantly preclinical stage of investigation [136].

Vaccine-based strategies, including dendritic cell-based vaccines and peptide vaccines, aim to induce antigen-specific immune responses against lesion-associated targets. By loading lesion-derived antigens or lysates onto mature dendritic cells, these approaches may activate both CD8^+^ and CD4^+^ T cell responses and enhance immune-mediated clearance of ectopic tissue. However, their translational potential is limited by challenges such as insufficient antigen specificity and concerns regarding reproductive safety, and robust clinical evidence is still lacking [138].

From a translational perspective, T cell-targeted therapies in endometriosis face substantial challenges. Current evidence from publicly available clinical trial registries and published studies indicates a lack of large-scale, well-designed clinical trials specifically targeting engineered T cells. Some exploratory immunotherapy trials (e.g., open-label studies such as NCT03340324) have been conducted; however, these generally do not involve T cell engineering strategies. In contrast, preclinical studies have demonstrated that modulation of T cell function—including Treg transfer, checkpoint blockade, and epigenetic or metabolic interventions—can reshape the immune microenvironment and suppress lesion progression. Nevertheless, these findings are largely derived from in vitro systems and animal models, and significant barriers remain for clinical translation [109,136].

### 3.6. Research Progress on Targeted B-Cells Therapies for Endometriosis

Histological and molecular studies have demonstrated the enrichment of plasma cells and deposition of immunoglobulins within endometriotic lesions, indicating the involvement of humoral immune responses. In addition, lesion-associated macrophages may support B cell survival and differentiation into plasma cells through the secretion of factors such as BAFF and B lymphocyte stimulator (BLyS), suggesting that activation of the local B cell–plasma cell axis contributes to the maintenance of the immunological microenvironment [91]. Consistently, multiple studies have reported the presence of auto-antibody profiles against endometrial-related antigens in patients with endometriosis, although variability exists across studies. Collectively, these findings support a role for B cells and antibody-mediated immunity in disease pathogenesis [93,140]. Furthermore, Mendelian randomization analyses based on genetic epidemiology have identified associations between immune cell-related traits and the risk of endometriosis, implicating B cell-related pathways in disease susceptibility [94].

B cell depletion strategies targeting CD20, such as rituximab, eliminate mature CD20^+^ B cells, thereby reducing de novo antibody production and impairing antigen presentation. However, as CD20 is not expressed on terminally differentiated plasma cells—particularly long-lived plasma cells—the impact of this approach on pre-existing humoral immunity is limited. In animal models of endometriosis, B cell depletion has been reported to reduce lesion size and attenuate fibrosis, although the evidence remains limited and heterogeneous across models. Importantly, the clinical application of such strategies raises safety concerns, including long-term immunosuppression, increased infection risk, and potential effects on reproductive function, leading to uncertainty regarding their translational applicability [113,141]. Although B cell-targeted therapies have been extensively used in oncology and autoimmune diseases with relatively well-characterized pharmacological profiles, direct clinical evidence in endometriosis is still lacking. Moreover, given their limited effects on plasma cell-derived antibodies, B cell depletion alone may be insufficient to fully suppress humoral immune responses, and combination strategies—such as co-targeting the BAFF pathway or plasma cells—may be required [113].

BAFF and BLyS are key regulators of B cell survival and differentiation. Elevated BAFF levels have been reported in lesion tissues and biological fluids of patients with endometriosis, suggesting a role in sustaining local B cell-mediated immune responses. Inhibition of BAFF signaling maybe reduce B cell survival and limit the generation of new antibodies, although it has minimal impact on antibodies produced by long-lived plasma cells [92,142]. Therapeutic agents targeting BAFF, such as belimumab, have demonstrated clinical efficacy in autoimmune diseases, including systemic lupus erythematosus. In the context of endometriosis, emerging evidence supports the involvement of the BAFF axis in local immune regulation; however, clinical studies targeting this pathway are currently lacking. Thus, BAFF signaling represents a promising yet unvalidated therapeutic target in this disease [142].

Bregs, particularly IL-10–producing subsets, have been reported to exhibit quantitative and functional alterations in endometriosis and may contribute to the establishment of an immunosuppressive microenvironment. Bregs display context-dependent roles, exerting anti-inflammatory effects under certain conditions while potentially promoting immune tolerance and lesion persistence in others. Therefore, therapeutic strategies targeting Bregs should focus on functional reprogramming rather than indiscriminate depletion. Given their context-specific effects, patient stratification based on immune phenotypes will be essential to minimize unintended immunological consequences [111]. From a translational perspective, B cell-targeted therapies in endometriosis remain at an early stage of development. Based on publicly available clinical trial registries, including ClinicalTrials.gov and WHO ICTRP, there is currently a lack of large-scale interventional studies specifically evaluating B cell-targeted agents in this disease. Representative drugs such as rituximab and belimumab have not yet been assessed in randomized controlled trials for endometriosis. Overall, this field is still transitioning from mechanistic exploration toward early-stage clinical translation.

## 4. Conclusions and Future Directions

Accumulating evidence indicates that immune dysregulation is a central mechanism underlying the initiation and progression of endometriosis. The disease is characterized by the establishment of a chronic inflammatory microenvironment within the peritoneal cavity, accompanied by functional alterations across multiple immune cell populations. In the peritoneal fluid of affected patients, innate immune cells—including macrophages, dendritic cells, and NK cells—exhibit significant changes in both abundance and activation status, highlighting the critical role of innate immunity in disease pathogenesis. Among these, macrophages represent the predominant immune cell population in the peritoneal niche and display increased abundance with a skewing toward an M2-like phenotype, which promotes angiogenesis, tissue remodeling, and lesion persistence [143,144]. Mechanistically, inflammatory mediators such as IL-17A have been shown to enhance macrophage recruitment and drive M2 polarization, thereby facilitating lesion establishment and progression [145]. In parallel, activation of the NLRP3 inflammasome in macrophages promotes the secretion of IL-1β, which in turn enhances the migratory capacity of endometrial stromal cells and contributes to ectopic lesion formation [146]. Beyond innate immunity, adaptive immune cells also play essential roles. Th17 cells and their signature cytokine IL-17 are significantly elevated in endometriosis and contribute to both inflammatory amplification and lesion maintenance [88,147]. Notably, IL-17A not only supports the survival of ectopic endometrial cells but also suppresses NK cell-mediated cytotoxicity, thereby facilitating immune evasion [147]. Concurrently, Tregs are enriched within endometriotic lesions and promote fibrosis and tissue remodeling through pathways involving TGF-β1 and platelet-derived growth factor (PDGF). Tregs further reinforce the immunosuppressive microenvironment by inducing M2 macrophage polarization and suppressing Th1 and Th17 differentiation [148]. Collectively, these findings underscore a complex network of immune cell interactions that shapes the immunological microenvironment of endometriotic lesions and drives disease progression. Accordingly, targeting immune cells and their associated signaling pathways represents a promising non-hormonal therapeutic strategy for endometriosis. Despite these advances, several limitations remain. First, substantial heterogeneity exists in the immune microenvironment among patients, as well as across different lesion types, leading to variability in immune cell composition and activation states [143]. Second, most mechanistic insights are derived from in vitro systems and animal models, with relatively limited clinical validation [145]. In addition, intricate crosstalk among immune responses, inflammatory signaling, and estrogen-dependent pathways further complicates the development of effective immunotherapies [148]. Therefore, more comprehensive and systematic studies are required to elucidate the precise mechanisms of immune regulation in endometriosis [144].

In addition to mechanistic limitations, the clinical translation of immunotherapy for endometriosis faces several important obstacles. To facilitate the clinical translation of immunomodulatory therapies, recent insights from MR studies offer a valuable supplementary perspective for drug discovery [149]. By leveraging genetic instruments to infer potential causality, MR studies suggest complex mediating networks among lipid metabolism, specific inflammatory proteins, and immune cell behaviors in endometriosis. Furthermore, genetic evidence highlights potential druggable targets; for instance, the implied role of UDP-Glucose Ceramide Glucosyltransferase (UGCG) inhibition in modulating immune pathways to influence disease risk. In the context of biologics, MR investigations into circulating cytokines can assist in evaluating systemic inflammatory markers that may have causal relevance. Collectively, integrating these genetic insights with traditional functional studies can help researchers better prioritize therapeutic targets and optimize the development pipeline for novel immunomodulators. Many immune pathways involved in lesion persistence also participate in physiological processes such as endometrial repair, embryo implantation, and reproductive immune tolerance. Consequently, systemic immune modulation may increase the risk of impaired fertility, abnormal pregnancy outcomes, infection, or autoimmune-related adverse effects. Furthermore, the marked heterogeneity of endometriosis—including differences in lesion subtype, hormonal milieu, inflammatory phenotype, and immune-cell composition—complicates patient stratification and limits the identification of universally effective therapeutic targets. Another major challenge is the lack of validated biomarkers for predicting therapeutic response and monitoring immune-related efficacy. In addition, most current studies remain restricted to animal models or small exploratory investigations, while large-scale multicenter clinical trials are still lacking. Technical issues related to local drug delivery, long-term safety evaluation, and optimization of treatment timing during the menstrual cycle also remain unresolved. Addressing these translational barriers will be essential for the successful development of clinically applicable immune-targeted therapies for endometriosis.

An important challenge in the clinical management of endometriosis is the high rate of postoperative recurrence, particularly in younger premenopausal women [150,151]. Although surgical excision effectively removes visible lesions and provides symptomatic relief, it does not eliminate the underlying hormonal and immune abnormalities that contribute to the establishment of new ectopic lesions [152]. Consequently, many patients require repeated surgical interventions or prolonged medical therapy during their reproductive years. In contrast, recurrence is substantially less common after menopause because of reduced estrogen stimulation, and complete lesion removal may therefore provide more durable disease control in selected patients. These characteristics highlight the need for immunomodulatory therapies that not only suppress lesion progression but also restore immune surveillance and prevent postoperative recurrence.

Future research should focus on high-resolution characterization of the immune microenvironment and its regulatory networks. Advances in single-cell technologies will enable the identification of key immune cell subsets and signaling pathways involved in disease pathogenesis. Therapeutic strategies aimed at modulating immune cell function—such as reprogramming macrophage polarization, restoring NK cell cytotoxicity, and rebalancing Th17/Treg responses—represent promising directions for intervention. Furthermore, multicenter clinical studies are needed to evaluate the efficacy of immunomodulatory therapies in terms of pain relief, disease recurrence, and reproductive outcomes.

## Figures and Tables

**Table 1:** Overview of immune cell–targeted therapeutic strategies for endometriosis.

Target	Key Targets/Pathways	Representative Strategies or Agents	Therapeutic Mechanism	Development Stage	Main Safety Concerns	Outcomes	Reference
Macrophages	PI3Kγ/PTEN pathway	PI3Kγ inhibitors	Reprogram M2 macrophages toward M1 phenotype	Preclinical	Off-target immune activation	Improved immune clearance	[103]
NK cells	KIR2DL1, NKG2A, LILRBs	Inhibitory receptor blockade	Restore NK-cell cytotoxicity	Early exploratory	Potential reproductive toxicity and autoimmunity	Restored NK cell cytotoxicity	[104]
NK cells	IL-2/IL-15 signaling	IL-2, IL-15	Enhance NK-cell activation and cytotoxicity	Preclinical/pilot clinical study	Cytokine-related toxicity	Improve NK cell activity	[104]
NK cells	Autologous NK-cell therapy	NK-cell infusion combined with GnRH agonists	Immune-mediated clearance of ectopic lesions	Clinical study	Endometrial receptivity impairment	Undergoing	[105]
Dendritic cells	IL-10 axis	Anti–IL-10 neutralizing antibodies	Reverse immunosuppressive signaling	Preclinical	Loss of immune tolerance	Suppressed angiogenesis	[70]
Dendritic cells	BDCA2	Anti-BDCA2 antibodies	Inhibit pDCs activation and recruitment	Preclinical	Immune dysregulation	Reduce immunosuppressive signals	[106]
Dendritic cells	TLR pathways	CpG-ODN, GM-CSF, FLT3L	Promote DCs maturation and Th1 responses	Preclinical	Excessive immune activation	Improve the immune response	[71]
Dendritic cells	IDO/STAT3	IDO inhibitors, STAT3 inhibitors	Reverse tolerogenic DCs phenotype	Preclinical	Systemic inflammatory toxicity	Undergoing	[71]
Neutrophils/NETs	IL-8/CXCR1/2	CXCR2 inhibitors	Reduce neutrophil recruitment and angiogenesis	Preclinical	Infection susceptibility	Suppressed angiogenesis	[107]
Neutrophils/NETs	PAD4/NET formation	PAD4 inhibitors, DNase	Suppress NET formation and inflammatory amplification	Preclinical	Impaired antimicrobial defense	Inhibiting the formation of NETs	[78]
Mast cells	IL-33/ST2 axis	IL-33/ST2 blockade	Reduce mast-cell activation, fibrosis, and EMT	Preclinical	Potential effects on implantation	Inhibiting the fibrotic process	[37]
Mast cells	Mast-cell degranulation	Cromolyn sodium, ketotifen	Stabilize mast cells and suppress mediator release	preclinical	Limited efficacy data	Reduce inflammation-related pain sensitivity	[108]
T cells	Treg modulation	Treg transfer or depletion	Rebalance inflammatory and suppressive immunity	Preclinical	Immune imbalance	Improve Treg capacity	[109]
B cells	CD20	Rituximab	Deplete mature B cells	Preclinical/extrapolated from autoimmune diseases	Long-term immunosuppression	Weakening the antigen-presenting function of B cells	[110]
B cells	Regulatory B cells	Functional reprogramming strategies	Modulate IL-10-mediated immune suppression	Exploratory	Context-dependent immune effects	Undergoing	[111]

**Table 2:** Clinical trials on immunotherapy for endometriosis.

ClinicalTrials.gov Identifier	Target/Focus	Drug/Biomarker	Study Design	Study Type	Phase	Status	Enrollment	Main Outcome	References
NCT03136978	Macrophages	M1 markers (CD14, CD68, CCR7, CD80) M2 markers (CD14, CD68, CD163, CD206)	Quantification of M1 and M2 macrophages in endometriotic tissue for each stage of endometriosis.	Observational	Not Applicable	Completed	45	Not robust enough to drawn firm conclusion	[123]
NCT04091997	Macrophage	Macrophage Migration Inhibitory Factor	Macrophage Migration Inhibitory Factor for Diagnosing Endometriosis and Its Severity	Observational	Not Applicable	Completed	300	Serum MIF is a promising marker for noninvasive diagnosis of severity of endometriosis	[124]
NCT07078435	Innate immune cells (include Macrophage) microbiota	Immune biomarkers	Immunologic and microbiota profiling in endometriosis	Interventional	Not Applicable	Not yet recruiting	40 (Estimated)	Ongoing	[125]
NCT01329796	Peritoneal inflammation (macrophage activity)	Lignocaine	Lignocaine pertubation for pain and inflammation control	Interventional	II	Completed	42	Unknown	[126]

## Data Availability

No new data were created or analyzed in this study. Data sharing is not applicable to this article.

## Abbreviations

The following abbreviations are used in this manuscript: Global Burden of Disease (GBD); disease-associated macrophages (DAMs) Vascular endothelial growth factor (VEGF); Dendritic Cells (DCs); Interleukin (IL); transforming growth factor-β (TGF-β); neutrophil extracellular traps (NETs); Plasmin-ogen Activator Inhibitor-1 (PAI-1); major histocompatibility complex class II (MHC II); interferon-γ (IFN-γ); tumor necrosis factor-α (TNF-α); lipopolysaccharide (LPS); Chemokine (C-X-C motif) ligand (CXCL8); nerve growth factor (NGF); estrogen receptor β (ERβ); C–C motif chemokine ligand 2 (CCL2); Monocyte Chemoattractant Protein-1 (MCP-1); nuclear factor kappa-B (NF-κB); Tumor-associated macrophages; (TAMs) Tumor Necrosis Factor Receptor 2 (TNFR2); B-cell lymphoma-2 (Bcl-2); Natural Killer Group 2D (NKG2D); Natural Killer Group 2 Member A (NKG2A); TNF-related apoptosis-inducing ligand (TRAIL); killer cell immunoglobulin-like receptors (KIRs); Killer cell lectin-like receptor D1 (CD94); Natural Killer cell protein 30 (NKp30); killer cell immunoglobulin like receptor, two Ig domains and long cytoplasmic tail 1(KIR2DL1); programmed cell death protein 1 (PD-1); human leucocyte antigen-G (HLA-G); human leucocyte antigen-E (HLA-E); killer cell immunoglobulin like receptor, two Ig domains and long cytoplasmic tail 4 (KIR2DL4); conventional dendritic cells (cDCs); myeloid dendritic cells (mDCs); plasmacytoid dendritic cells (pDCs); Cluster of Differentiation 80 (CD80); Cluster of Differentiation 86 (CD86); prostaglandin E_2_ (PGE_2_); indoleamine 2,3-dioxygenase (IDO); 11β-hydroxysteroid dehydrogenase type 1 (HSD11B1); granulocyte colony-stimulating factor (G-CSF); matrix metalloproteinases (MMPs) neutrophil elastase (NE); reactive oxygen species (ROS); epithelial–mesenchymal transition (EMT); damage-associated molecular patterns (DAMPs); Toll-like receptor 4 (TLR4); Toll-like receptor 2 (TLR2); major basic protein (MBP); prostaglandin D_2_ (PGD_2_); innate lymphoid cells (ILC2s); janus kinase Signal Transducer and Activator of Transcription 3 (JAK–STAT); interleukin 1 receptor-like 1 (IL-1RL1); T helper cell 17 (Th17); Regulatory T cells (Treg); Cluster of Differentiation 4 (CD4^+^); Lymphocyte activation gene 3 (LAG-3); T cell immunoglobulin and mucin-domain containing protein 3 (TIM-3); Programmed Cell Death Ligand 1 (PD-L1); Cytotoxic T-Lymphocyte Antigen 4 (CTLA-4); mitogen-activated protein kinase (MAPK); immunoglobulin (IgM/IgG); B-cell activating factor (BAFF, also known as BLyS); transmembrane activator and calcium modulator and cyclophilin ligand interactor (TACI); B-cell maturation antigen (BCMA); regulatory B cells (Breg); B cell receptor (BCR); Phosphatidylinositol 3-Kinase/Protein Kinase B Signaling Pathway (PI3K–AKT); endometriotic microenvironment (EME); Mendelian randomization (MR); UDP-Glucose Ceramide Glucosyltransferase (UGCG); macrophage colony-stimulating factor (M-CSF); leukocyte immunoglobulin-like receptor B1 (LILRB1); leukocyte immunoglobulin-like receptor B2 (LILRB2); gonadotropin-releasing hormone (GnRH); Blood Dendritic Cell Antigen 2 (BDCA2); mammalian target of rapamycin (mTOR); immunohistochemistry (IHC); Cytosine–Phosphodiester–Guanine (CpG); FMS-like tyrosine kinase 3 ligand (FLT3L); Deoxyribonuclease I (DNase); peptidylarginine deiminase 4 (PAD4); retinoic acid-related orphan receptor γt (RORγt); chimeric antigen receptor T cells (CAR-T); T cell receptor-engineered T cells (TCR-T); platelet-derived growth factor (PDGF).
